# Microencapsulation of *Lactobacillus plantarum* with Improved Survivability Using Pufferfish Skin Gelatin-Based Wall Materials

**DOI:** 10.3390/md22030124

**Published:** 2024-03-05

**Authors:** Honghui Guo, Yelin Zhou, Quanling Xie, Hui Chen, Yiping Zhang, Zhuan Hong, Sijin Chen, Ming’en Zhang

**Affiliations:** 1 Engineering Technology Innovation Center for the Development and Utilization of Marine Living Resources, Third Institute of Oceanography, Ministry of Natural Resources, Xiamen 361005, Chinahysschenhui@tio.org.cn (H.C.); ypzhang@tio.org.cn (Y.Z.); sjchen@tio.org.cn (S.C.); 18558969140@163.com (M.Z.); 2 Xiamen Ocean Vocational College, Xiamen 361100, China; 3 Fujian Key Laboratory of Island Monitoring and Ecological Development, Island Research Center, Ministry of Natural Resources, Fuzhou 350400, China; 4 College of Advanced Manufacturing, Fuzhou University, Quanzhou 362200, China

**Keywords:** microcapsule, pufferfish skin, gelatin, sodium caseinate, *Lactobacillus plantarum*, spray-drying

## Abstract

To improve the survivability of probiotics, *Lactobacillus plantarum* was microencapsulated using pufferfish skin gelatin (PSG)-based wall materials by spray-drying. This work investigated the protective effect of three different pH-dependent proteins (sodium caseinate (SC), soy protein isolate (SPI), and whey protein isolate (WPI)) combined with PSG on *L. plantarum*. The experimental results of spray-drying with an inlet temperature of 120 °C and an outlet temperature of 80 °C, storage at 4 °C for 6 months, simulated digestion, and turbidity indicated that PSG/SC had better stability and encapsulation effects and was more suitable to encapsulate *L. plantarum* than PSG/SPI and PSG/WPI. The optimum preparation conditions for *L. plantarum* microcapsules were a PSG/SC mass ratio of 2:1, an SC concentration of 20 g/L, and a cell concentration of 10 g/L. The encapsulation efficiency of the obtained microcapsules was 95.0%, and the survival rate was 94.2% in simulated gastric fluid for 2 h and 98.0% in simulated intestinal fluid for 2 h. Amino acid composition analysis exhibited that the imino acid and aspartic acid contents of PSG were 27.98 and 26.16 g/100 g protein, respectively, which was much higher than commercial bovine gelatin. This characteristic was favorable to the high encapsulation efficiency and stability of microcapsules. In vitro release experiments showed that the PSG/SC microcapsules did not disintegrate in simulated gastric fluid for 2 h but could completely release in simulated intestinal fluid for 2 h, which can maintain the high survivability of *L. plantarum* in simulated digestion. In general, this study demonstrated that microcapsules using PSG/SC as wall materials can effectively improve the survivability of probiotics and have great potential for application in probiotic products.

## 1. Introduction

Probiotics have been defined as ‘live microorganisms that, when administered in adequate amounts, confer a health benefit on the host’ [1]. To exert their biological functions, the number of viable bacteria in orally administered probiotics should reach at least 10^6^ CFU/g [2]. However, most probiotics cannot meet this recommended level at the intestinal target site because environmental stresses such as elevated temperature, the acidic pH of gastric juice, and digestive enzymes during processing, storage, and consumption reduce the viability of probiotics [2]. Hence, improving the survivability of probiotics is essential for the targeted delivery of sufficient viable bacteria to the host.

Microencapsulation is considered one of the most effective methods to improve the survivability of probiotics. Live microorganisms are embedded in tiny capsule-like particles that can provide a physical barrier to resist adverse environmental conditions. Microencapsulation can also control and target the release of active ingredients or mask undesirable tastes and flavors [3]. At present, the microencapsulation methods include extrusion, emulsification, spray-drying, freeze-drying, and spray chilling, each of which has advantages and disadvantages [4]. Generally, spray-drying and freeze-drying are the main applied technologies. The freeze-drying technique involves the removal of water by the sublimation process at a reduced pressure while having the advantage of minimal heat stress on probiotics [5]. However, very low freezing temperatures may also lead to the inactivity of probiotics. In addition, freeze-drying has a high cost and energy consumption, and it is difficult to control the particle size. Spray-drying is a crucial and prevalent microencapsulation technique that is efficient, simple, continuous, and cost-effective [6]. But the spray-drying technique is unfriendly to probiotics owing to its high temperature and rapid dehydration [7]. It is worth noting that in recent years, spray-drying has boomed in probiotic encapsulation with the enhancement of wall material properties [8].

The selection of wall material has an enormous impact on the stability and properties of microcapsules [9]. Several materials, such as polysaccharides (alginate, chitosan, gums, celluloses, etc.), proteins (gelatine, milk proteins, soy proteins, etc.), and fats, are used as encapsulants for probiotics [4,10]. In this regard, proteins are popular wall materials due to their high safety, cheapness, and favorable physicochemical properties [11]. Among these, gelatin is a good alternative because of its low acquisition cost, biodegradability, and biocompatibility, and its structure allows multiple combinations of molecular interactions, providing reasonable stability [12]. So far, most gelatin is produced from mammalian skin or bone [13]. As an alternative, the fish and their by-products appear as sources of collagen for obtaining gelatin. Fish skin contains a high amount of collagen. Using fish skin gelatin can solve the problem of fish waste disposal from the fish processing industry and create value-added products [14]. Fish skin gelatin has received more attention due to its advantages in safety and acceptability compared with mammalian source gelatin [15]. One promising material that can be used as a gelatin source is pufferfish skin. Pufferfish is a popular finfish species for aquaculture in East Asia because of its excellent taste and abundant nutrients [16]. The rapid growth of the pufferfish processing industry has provided many pufferfish skins that could be used for gelatin production [15].

*Lactobacillus plantarum* has a variety of biological functions, such as inhibiting the growth of harmful bacteria, improving the intestinal environment and immune regulation, maintaining the balance of blood glucose, lipids, and blood pressure, and regulating metabolic disorders. Thus, it has been associated with health benefits in bowel function, immune health, cardiovascular health, etc. [10,17]. However, *L. plantarum* is very sensitive to industrial processing, storage, and digestion, which markedly limits its use [10]. Hao et al. observed that the viable count of *L. plantarum* FZU3013 was reduced by >4 log CFU/mL during 5 weeks of storage at 4 °C and was reduced by 6.6 CFU/mL after incubation in simulated gastric fluid (SGF, pH 2.0) for 60 min [18]. Guo et al. found that the cell viability of *L. plantarum* CICC 21805 decreased by ~9 log CFU/mL within 1 min at 85 °C [19].

In order to improve the survivability of *L. plantarum*, pufferfish skin gelatin (PSG) was combined with sodium caseinate (SC), whey protein isolate (WPI), and soy protein isolate (SPI), respectively, and used as wall material for the microencapsulation of *L. plantarum* by spray-drying. The physicochemical properties of the spray-dried microcapsules in terms of morphology, structure, encapsulation efficiency, survival rate, and in vitro release were investigated. Meanwhile, PSG was compared with commercial bovine gelatin (BG) to illustrate the effect of gelatin species on microcapsule performance. Therefore, the potential of PSG as a wall material for probiotic microencapsulation was evaluated, assessing the survivability of microencapsulated probiotics under simulated gastrointestinal conditions and during storage. This work will be beneficial for understanding the effect of protein-based wall material combinations on microcapsule function and provide helpful information on the high-value development of aquatic by-products, indicating the potential application of PSG-based probiotic microcapsules in the food and pharmaceutical fields.

## 2. Results and Discussion

### 2.1. Effects of Wall Materials on Survivability and Storage Stability

Table 1 shows the effect of different wall materials on the viability of *L. plantarum* during spray-drying. The initial viable count of *L. plantarum* was about 9 log CFU/g. After spray-drying, the viable count of free cells decreased to 2.1 log CFU/g, while the viable count of *L. plantarum* microencapsulated with PSG/SC, PSG/SPI, and PSG/WPI was about 7.7, 5.2, and 6.9 log CFU/g, respectively. The decrease in viability was due to the rapid evaporation of water and high temperatures during the spray-drying process. Similar results were reported by Moumita et al., who mentioned that outlet temperatures above 75 °C would result in the drastic inactivation of bacteria [20]. Although the high temperature of spray-drying can cause severe damage to the microorganisms [7], the wall materials of PSG/SC, PSG/SPI, and PSG/WPI exhibited good high-temperature protection for bacteria. These PSG-based wall materials may be related to reducing heat transfer because of hydrophobic amino acid residues in the composite protein structure [21,22]. Viscosity may be another factor affecting the heat transfer rate. Arslan reported a similar result that gelatin as a wall material in *S. boulardii* microencapsulation provided better survivability than five other different wall materials (whey protein concentrate, modified starch, maltodextrin, pea protein isolate, and gum Arabic) during the spray-drying process due to its effect on reducing heat transfer [21]. Moreover, SC is a heat-tolerant protein, which is more beneficial for reducing the heat transfer of spray-drying [23]. Hence, compared with PSG/SPI and PSG/WPI, PSG/SC can better improve the survivability of *L. plantarum* during spray-drying.

**Figure 1:**
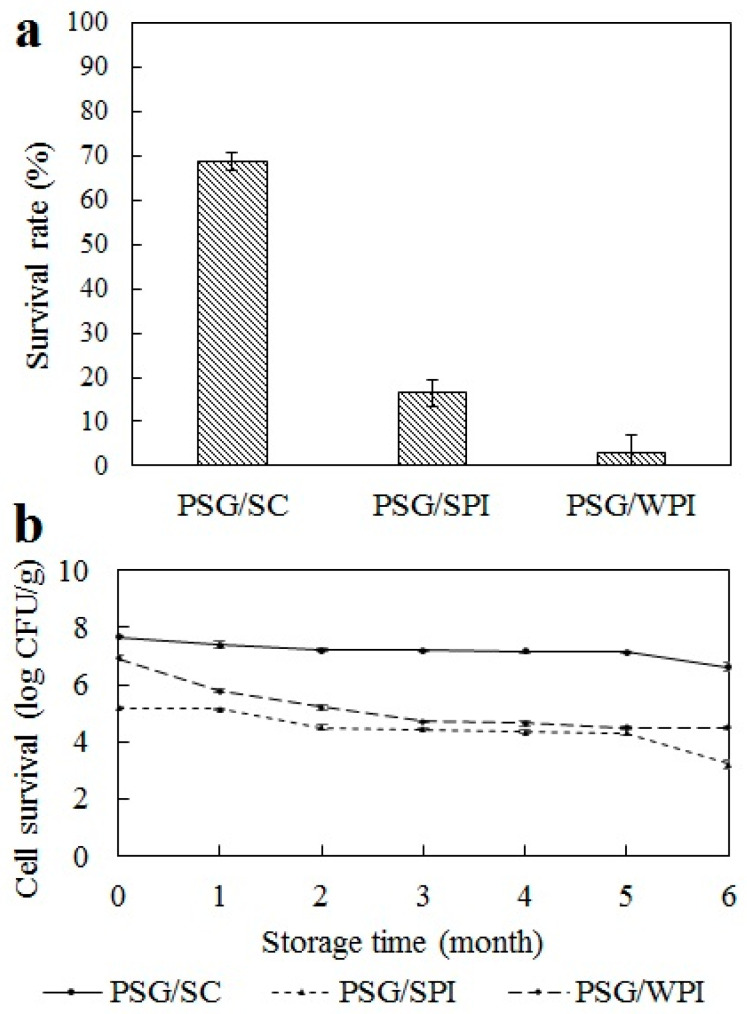
(**a**) Survival rate of *L. plantarum* microencapsulated with PSG/SC, PSG/SPI, and PSG/WPI after incubation in SGF for 2 h. (**b**) Survivability of *L. plantarum* microencapsulated with PSG/SC, PSG/SPI, and PSG/WPI during storage at 4 °C for 6 months. The results are indicated as mean ± standard deviation (*n* = 3).

The acidity and pepsin of SGF can severely impair *L. plantarum*. After incubation in SGF for 2 h, the viable count of free cells declined from about 9 log CFU/mL to 1 log CFU/mL. However, as shown in Figure 1a, the survival rate of *L. plantarum* microencapsulated with PSG/SC, PSG/SPI, and PSG/WPI in SGF was significantly higher than that of free cells. The results suggested that the survivability of *L. plantarum* was affected considerably by wall materials. The protein-based wall material has a less polar group, like hydroxyl, than polysaccharides, which can keep the acid effect out of the microcapsule core [21]. Furthermore, *L. plantarum* microencapsulated with PSG/SC showed a higher survival rate than PSG/SPI and PSG/WPI.

As shown in Figure 1b, the viable count of *L. plantarum*-loaded PSG/SC microcapsules decreased from 7.7 to 6.8 log CFU/g during the 6-month storage period; the logarithmic reduction in viable *L. plantarum* was about 0.9, which showed an apparent decline after about 5 months. The viable count of *L. plantarum*-loaded PSG/SPI microcapsules started to decline significantly within the first month of storage and decreased from the initial 5.2 to 3.2 log CFU/g after 6 months, with a logarithmic reduction of 2 log CFU/g. The viable count of *L. plantarum*-loaded PSG/WPI microcapsules decreased from 6.9 to 4.5 log CFU/g. It was clear that PSG/SC provided better protection for cells than PSG/SPI or PSG/WPI during storage. SC is a hydrophobic protein, which provides a more hydrophobic barrier for *L. plantarum* than SPI and WPI during storage [23]. Additionally, the heteroprotein interaction of PSG/SC may also affect the structural and physical properties of microcapsules to enhance the survivability of *L. plantarum*.

### 2.2. Effect of Wall Materials on Turbidity

**Figure 2:**
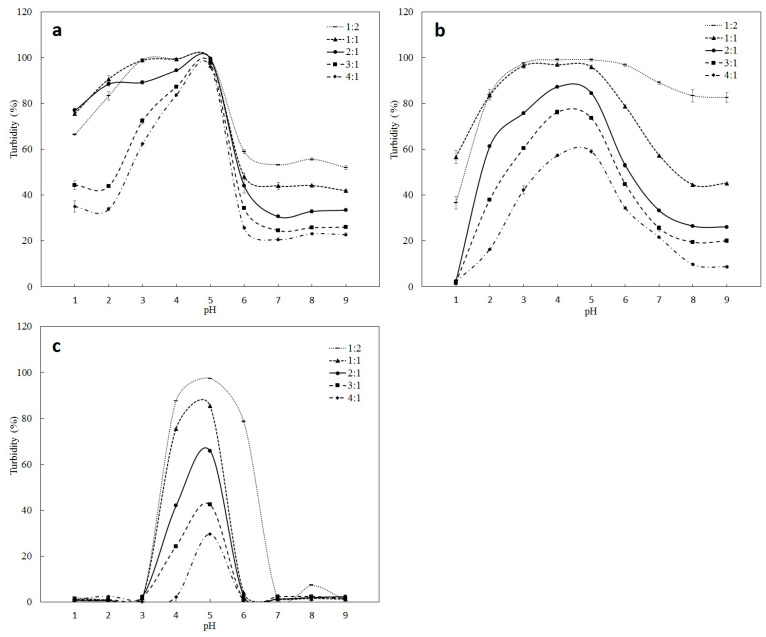
Turbidity measurement of (**a**) PSG/SC, (**b**) PSG/SPI, and (**c**) PSG/WPI microcapsule solutions at five different mixing ratios (1:2, 1:1, 2:1, 3:1, and 4:1) as a function of pH. The results are indicated as mean ± standard deviation (*n* = 3).

When polymer materials are dispersed in aqueous media, the solvent pH influences electrostatic interactions among polymers, affecting the number of charged reactive groups on the polymer surface [24]. The turbidity value can reflect electrostatic interactions among polymers. Figure 2a shows the effects of pH and the mixing ratio of PSG/SC on turbidity. The turbidity of the PSG/SC microcapsule solution at different mixing ratios reached an apex at about pH 5.0. The isoelectric point (pI) of SC is 4.6 [25], while the pI value of PSG is measured at about 6.0. Therefore, the pH value of 5.0 was between the pI of SC and PSG, where electrostatic attraction occurred between anionic SC molecules and positively charged PSG molecules. The neutralization of opposite charges led to the formation of insoluble sediment and the maximum turbidity [26]. For PSG/SPI (Figure 2b) and PSG/WPI (Figure 2c), the maximum value of turbidity was also obtained at pH 5.0, mainly because the pI value of SPI and WPI was about 4.6, similar to that of SC. The turbidity results of PSG/SC, PSG/SPI, and PSG/WPI were significantly influenced by the mixing ratio of materials, which were considered crucial factors in maintaining the charge balance and the intensity of interactions among polyions. Hamid et al. also reported that the turbidity was affected by the volume ratio of cress seed mucilage (CSM) to SC. The maximum turbidity occurred at a CSM/SC volume ratio of 1:2 [25].

When the pH value decreased from 5 to 3, the turbidity at PSG/SC mixing ratios of 1:2 and 1:1 exhibited higher values than other mixing ratios. When the pH value was between 1 and 2, the maximum turbidity was obtained at PSG/SC mixing ratios of 1:1 and 2:1. A high value of turbidity below pH 4 may be due to the protonation of a large number of carboxylic groups on PSG and SC molecules [24]. PSG contains abundant acidic amino acids, such as aspartic acid and glutamic acid [22]. Moreover, the weak charge density of positively charged PSG/SC is not enough to overcome the gravity effect of the aggregates, resulting in turbidity. For PSG/SPI (Figure 2b) and PSG/WPI (Figure 2c), the turbidity value decreased as the pH value was adjusted from 5 to 1. The PSG/SPI solution at 1:2 and 1:1 mixing ratios was non-transparent at pH 3~5, and the turbidity value was 95~99%. Then, while further lowering the pH value, the turbidity decreased dramatically. At pH values between 1 and 2, the turbidity value was lower than that of the PSG/SC solution, and the solution became more transparent. However, the turbidity value of the PSG/WPI solution at different mixing ratios was in the range of 0~3% at pH 1~2, and the solution was completely transparent. The results might be attributed to the increasing number of positively charged amino groups as the pH decreased [24]. The strong electrostatic repulsion between similarly charged PSG and SPI/WPI molecules led to the declining turbidity value.

It was observed that the turbidity of PSG/SC, PSG/SPI, and PSG/WPI microcapsule solutions decreased as the pH value increased from 5 to 9. The turbidity of the PSG/SC microcapsule solution at different mixing ratios appeared to have a relatively low value above pH 6, which can be related to the electrostatic repulsion between PSG and SC due to their anionic natures [25]. Turbidity results indicated that the PSG/SC microcapsules at a mixing ratio of 2:1 exhibited high turbidity at pH 1~2 and low turbidity under neutral conditions. It shows a good prospect for applying the microcapsule as a gastrointestinal delivery carrier. 

Therefore, based on the results of spray-drying, storage, simulated digestion, and turbidity, PSG/SC was chosen to encapsulate *L. plantarum* for subsequent experiments.

### 2.3. Effects of PSG/SC Mass Ratio, SC Concentration, and L. plantarum Concentration on Survival Rate and Encapsulation Efficiency

**Figure 3:**
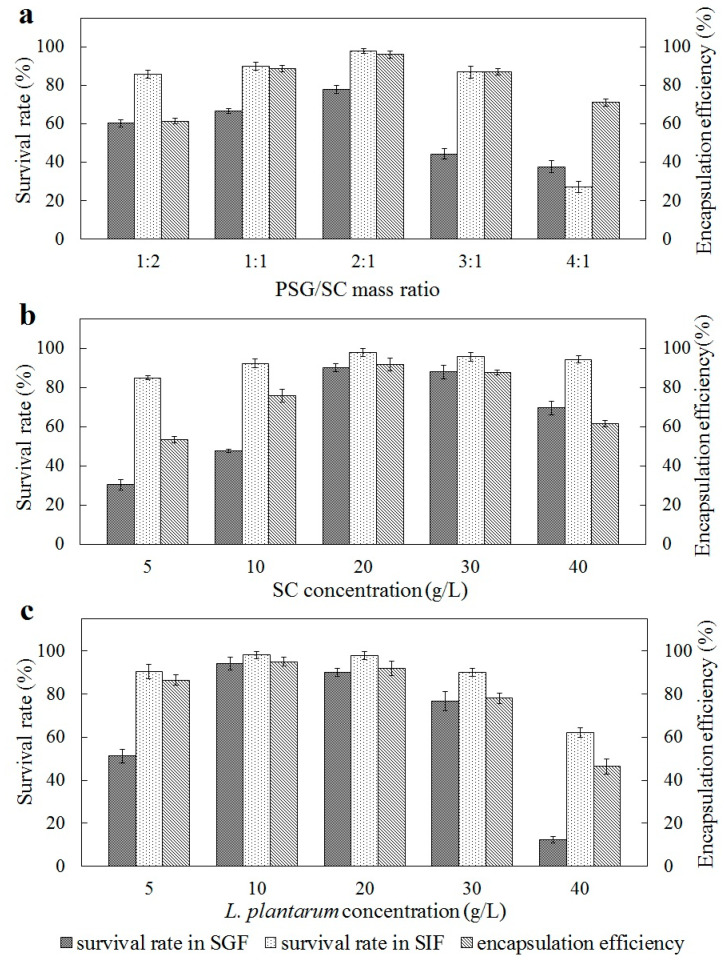
Effects of PSG/SC mass ratio, (**a**) SC concentration (**b**) and *L. plantarum* concentration (**c**) on survival rate in simulated gastrointestinal fluid and encapsulation efficiency. The results are indicated as mean ± standard deviation (*n* = 3).

The effects of various conditions during the microencapsulation process on survival rate and encapsulation efficiency are shown in Figure 3. After microencapsulation with different PSG/SC mass ratios, the survival rate of *L. plantarum* cells in both SGF and simulated intestinal fluid (SIF) was significantly improved, as shown in Figure 3a. Among the various ratios, PSG/SC microcapsules at a mixing ratio of 2:1 (*w*:*w*) provided the best protection for *L. plantarum* in SGF and SIF. The encapsulation efficiency and survival rate at this ratio were significantly higher than those of the other four mixing ratios (*p* < 0.05). Furthermore, the result was in good agreement with the turbidity tests. However, the survival rate of *L. plantarum* in PSG and SC monolayer microcapsules in SGF was about 1.28% and 0.16%, with encapsulation efficiency of 84.6% and 85.1%, respectively. Hence, the wall material of PSG or SC alone could not provide good protection for *L. plantarum* cells during the incubation in SGF. When exposed to SGF, PSG/SC at an optimal ratio of 2:1 caused the most efficient coacervate formation by modulating the charge balance among polyions and the intensity of interactions [25]. The polymeric network of PSG/SC microcapsules can protect *L. plantarum* cells from the permeability of H^+^ and pepsin, which helps sustain the integrity of the microcapsules [27]. Jiang et al. also found that the protein-based microcapsules appeared to have a more uniform structure to hold and shelter probiotic microorganisms and might be more resistant to enzymatic degradation in the simulated gastrointestinal tract than sodium alginate [28].

In Figure 3b, the encapsulation efficiency and survival rate rose with the increasing SC concentration. However, when the SC concentration was 40 g/L, the encapsulation efficiency and survival rate of SGF were significantly decreased. This was due to the decline in SC solubility at a high concentration above 30 g/L. Consequently, a uniform emulsion was challenging to form, which caused the low encapsulation efficiency of *L. plantarum*. This is similar to the results of Sun et al., who reported that the high concentration of soy protein isolate made it challenging to form a homogeneous solution and thereby caused the poor encapsulation number and viability of bacteria in digestive fluids [29]. No significant differences in cell loss at most concentrations of SC were observed during the 2 h incubation in SIF (*p* > 0.05).

As shown in Figure 3c, the best selection for *L. plantarum* concentration was 10 g/L. At a concentration of *L. plantarum* below 10 g/L, the lack of core material caused the formation of many empty microcapsules. When the *L. plantarum* concentration was above 30 g/L, a large number of free cells were exposed outside the microcapsules, resulting in the decline of encapsulation efficiency and the low survival rate of SGF [30]. Overall, the maximum encapsulation efficiency (95.0%), survival rate in SGF (94.2%), and in SIF (98.0%) were obtained based on the optimum preparation conditions, and they were a PSG/SC mass ratio of 2:1, an SC concentration of 20 g/L, and a *L. plantarum* concentration of 10 g/L.

### 2.4. Effect of Gelatin Species on Microcapsule Performance

There are differences in the properties of gelatin species, such as amino acid composition, sequence, and isoelectric point [31]. The encapsulation efficiency of *L. plantarum* microcapsules prepared by BG/SC was 87.0%. The survival rate of *L. plantarum* was 12.5% and 42.4% in SGF and SIF, respectively. Compared with PSG/SC, *L. plantarum* microcapsules prepared by BG/SC had a lower encapsulation efficiency and a poor protective effect on *L. plantarum* in the gastrointestinal fluid. The results showed that gelatin species could significantly affect the performance of microcapsules. Amino acid composition and surface charge were considered the main influencing factors. 

The amino acid composition of BG and PSG is presented in Table 2. Eighteen kinds of amino acids could be detected in both BG and PSG. The imino acid (proline and hydroxyproline) and glycine content of BG were 23.47 and 19.65 g/100 g of protein, respectively. The alanine acid of BG was 7.58 g/100 g of protein. However, the imino acid and glycine contents of PSG were 27.98 and 13.67 g/100 g of protein, respectively. The alanine content of PSG was 7.17 g/100 g of protein. The imino acid content of PSG was not only higher than that of BG but also higher than that of bigeye snapper, sin croaker, shortfin scad, seabass, and croaker, which were 14.4, 11.0, 10.0, 23.3, and 17.9 g/100 g protein [32]. Imino acid and glycine are important for gel strength, while alanine plays an essential role in providing the visco-elastic properties of gelatin [32,33]. Therefore, PSG had a high composition of these amino acids and showed excellent gelling power, which was helpful in forming stable microcapsules. The hydroxyproline content of PSG was 24.09 g/100 g of protein, much higher than that of BG (10.11 g/100 g of protein), which could affect the thermal stability of gelatin [32]. The above may lead to a higher encapsulation efficiency of microcapsules prepared by PSG than that prepared by BG. Besides imino acid, the aspartic acid of PSG was 26.16 g/100 g of protein, which is much higher than the 6.67 g/100 g of protein of BG. The presence of a large number of acidic amino acids has a significant influence on the surface charge characteristics of microcapsules.

**Figure 4:**
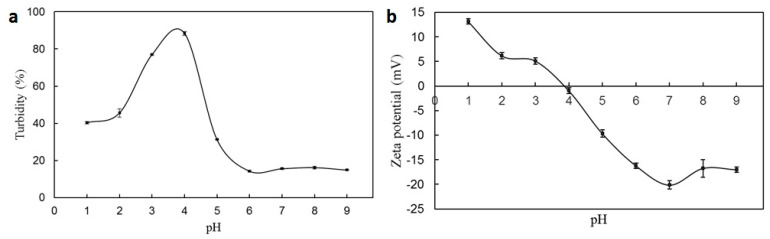
(**a**) Turbidity and (**b**) zeta potential of BG/SC microcapsule solution as a function of pH (data shown were the mean ± SD, *n* = 3).

Figure 4a shows the effects of pH on the turbidity of BG/SC microcapsules. The pI value of BG was measured at about 3.0, while the maximum turbidity of the BG/SC solution was obtained around pH 4.0, between the pI of BG and SC. At pH 1~2, the turbidity value of the BG/SC solution was in the range of 40~50%, lower than that of the PSG/SC solution. The BG/SC solution exhibited low turbidity values of 10~20% when the pH was 6~9. The zeta potential charge profile of BG/SC microcapsules is shown in Figure 4b. It could be seen that the electrical equivalence point of pH occurred at about 3.8, which matched well with the turbidity result. At the pH of SGF (pH 1.2), BG/SC microcapsules carried a positive charge, and the absolute zeta potential was greater than 10 mV, which was unfavorable to macromolecule aggregation and led to the loosening of microcapsules. Therefore, BG/SC microcapsules showed a poor protective effect on *L. plantarum* in SGF compared with PSG/SC microcapsules. This might be because the proportion of acidic amino acids in BG was much lower than that in PSG, and there were no large number of carboxyl groups to neutralize hydrogen ions [34]. At pH 6~9, the high absolute zeta potential of BG/SC microcapsules resulted in a strong electrostatic repulsive force between negatively charged macromolecules. Meanwhile, the BG/SC microcapsules disintegrated completely, and the solution became transparent. Based on the turbidity and zeta potential measurements, it can be concluded that the PSG/SC microcapsule has a better-controlled release effect in gastrointestinal fluid than the BG/SC microcapsule and is more suitable for embedding probiotics.

### 2.5. Characterization of Optimized PSG/SC Microcapsules

#### 2.5.1. SEM, TEM, and Particle Size Distribution

**Figure 5:**
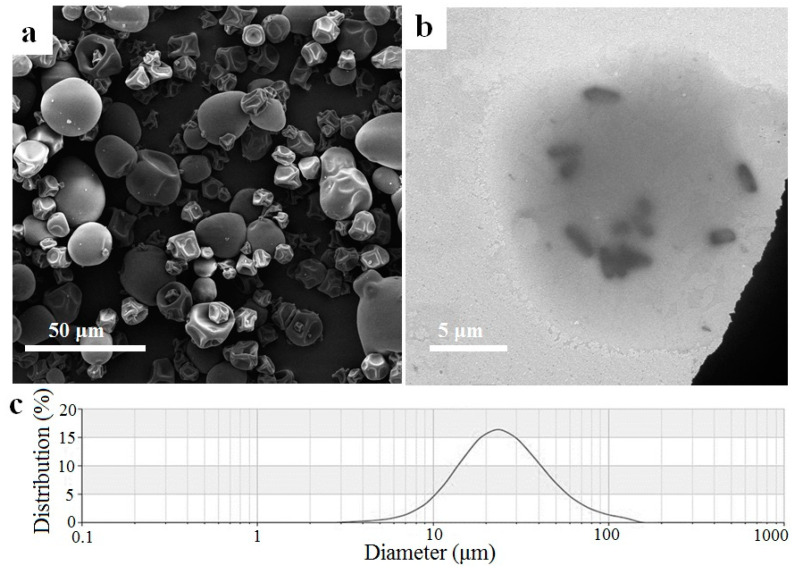
(**a**) SEM of *L. plantarum*-loaded microcapsules. (**b**) TEM of a single *L. plantarum*-loaded microcapsule. (**c**) Particle size distribution of *L. plantarum*-loaded microcapsules.

The SEM of *L. plantarum*-loaded PSG/SC microcapsules prepared with the optimal encapsulation parameters is presented in Figure 5a. The particle size distribution of microcapsules was 10~40 µm, and no bacteria were observed on the surface of the microcapsules. The shape of the particles was nearly spherical. The surface of the particles without notable cracks was mostly wrinkled with concavities, which can be attributed to the shrinkage of the particles caused by rapid evaporation of the water during spray-drying [11]. The inner structure of a single *L. plantarum*-loaded microcapsule was observed by TEM, as shown in Figure 5b. It can be seen that the diameter of a typical *L. plantarum*-loaded microcapsule was around 20~30 µm, and the rod-shaped bacteria were embedded entirely within the PSG/SC matrix. There was no interspace between bacterial cells and the PSG/SC matrix, reflecting the overall hydrophilic nature of the bacteria’s surfaces [35]. TEM observation indicated that the encapsulating effect of the PSG/SC microcapsule was excellent. The analysis results of the particle size distribution (Figure 5c) showed that the mean diameter of the microcapsules was about 22.8 µm (Span 1.2), almost consistent with those measured by SEM and TEM.

The temperature and feed flow during spray-drying can critically affect the microstructure of the microcapsules [36]. In this study, decreasing the outlet temperature and accelerating the feed flow may cause a high residual moisture content of powder, leading to a large size and agglomeration of particles. However, different PSG/SC mass ratios did not affect the diameter of microcapsules. This can be ascribed to the same parameters applied in spray-drying. A similar result was also observed in spray-dried *Lactobacillus* microcapsules using SC and fat as encapsulation materials [35]. The size of the microcapsules obtained in this study was in agreement with the value of <100 μm proposed in the studies of spray-dried probiotic microcapsules [37]. Compared with the probiotic microcapsules prepared by the extrusion method with a large size (mostly >1 mm) [4] and those prepared by the freeze-drying method with an irregular shape and uncontrollable size [5,38], the spray-drying microcapsules with a smaller size (<100 μm) and a more regular shape do not affect the texture of the food to which they are incorporated, and are preferred for most applications [37]. 

#### 2.5.2. FTIR Analysis

**Figure 6:**
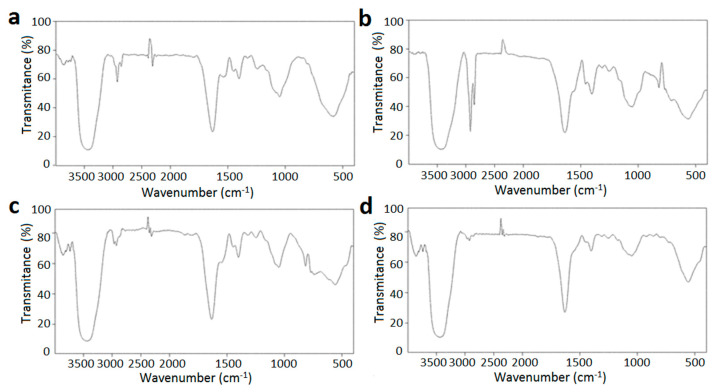
FTIR spectra of (**a**) PSG, (**b**) SC, (**c**) empty PSG/SC microcapsules, and (**d**) *L. plantarum*-loaded PSG/SC microcapsules.

In Figure 6, the FTIR spectra refer to PSG, SC, empty PSG/SC microcapsules, and *L. plantarum*-loaded PSG/SC microcapsules. The spectra of PSG and SC were analyzed as they were the primary wall materials. The absorption band of PSG at 3435.6 cm^−1^ was attributed to O-H stretching vibration. The absorption peak at 3049.4 cm^−1^ was mainly caused by N-H stretching vibration. The peaks at 2925.7 cm^−1^ and 2858.2 cm^−1^ belonged to the asymmetric and the symmetric stretching vibration of C-H, respectively. The spectrum of PSG showed two significant peaks attributed to the amide I and amide II of protein structure. The absorption peak at 1633.7 cm^−1^ mainly comes from the C=O stretching vibrations of the amide I band, while the C-N stretching and N-H bending vibrations of the amide II band caused the absorption peak at 1539.3 cm^−1^ [24]. The bands at 1446.7 cm^−1^ and 1403.1 cm^−1^ were attributed to C-H bending deformation vibration and the symmetric deformation vibration of -CH_3_, respectively. The peak at 1050.7 cm^−1^ corresponded to the stretching vibration of C-O.

The O-H stretching vibration of SC appeared at 3430.9 cm^−1^. The absorption peaks of SC at 2922.7 cm^−1^ and 2855.3 cm^−1^ were related to the asymmetric and symmetric stretching vibrations of C-H, respectively. The characteristic peak was assigned to the amide I at 1639.4 cm^−1^ for the stretching of the carbonyl group [25]. The C-H and -CH_3_ deformation vibrations were also included in the spectrum of SC, which were at 1456.9 cm^−1^ and 1404 cm^−1^, respectively. The -C-NH_2_ stretching vibration of the amide III band was seen at 1314.4 cm^−1^. The band at 1247.3 cm^−1^ and 1051.7 cm^−1^ referred to the stretching vibration of C-O-C and C-O [39].

Figure 6c shows the FTIR spectrum of empty PSG/SC microcapsules. It can be seen that the characteristic peaks of PSG and SC were detected in this spectrum. When PSG was combined with SC, the O-H stretching vibration was shifted to 3442.3 cm^−1^, and the C-H stretching vibration was shifted to 2966.5 and 2927.4 cm^−1^, respectively. These apparent shifts were caused by the formation of hydrogen bonds [27]. The interaction between *L. plantarum* and wall materials during the formation of cell-loaded microcapsules was observed in Figure 6d. The main absorption peaks of PSG and SC were also observed in this spectrum. However, the distinct bands corresponding to amide and phosphate groups from the probiotic bacteria were not observed. This could be attributed to the high microencapsulation efficiency of *L. plantarum* and the absence of bacteria on the surface of the microcapsules [11]. A broad band of the O-H group was shifted to 3439.1 cm^−1^, suggesting that hydrogen bonding might play an essential role in the formation of biocomposite [27]. The stretching vibration peaks of amide I and III bands were shifted to 1631.7 cm^−1^ and 1308.2 cm^−1^, respectively. Meanwhile, the peak assigned to the stretching vibration of C-O was shifted to 1253.4 cm^−1^. Therefore, analysis of FTIR features indicated that the formation of *L. plantarum*-loaded microcapsules was based on hydrogen and Van der Waals bonds, and no covalent bond was formed [11].

#### 2.5.3. Zeta Potential

**Figure 7:**
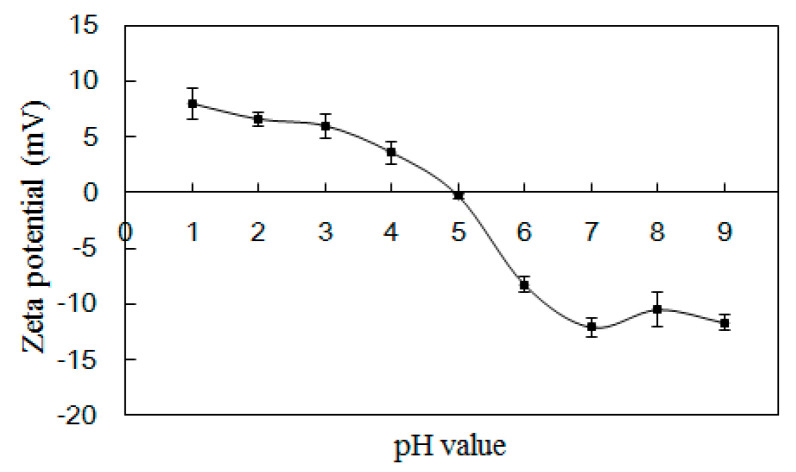
Zeta potential measurement of PSG/SC microcapsules without *L. plantarum* at different pH values from 1 to 9. The results are indicated as mean ± standard deviation (*n* = 3).

The zeta potential charge profile of PSG/SC microcapsules at a mixing ratio of 2:1 is shown in Figure 7. The electrical equivalence point pH occurred around 5.0, which was in accordance with the turbidity results (Figure 2a). As the pH was below 5.0, the PSG/SC microcapsules were positively charged. The low potential value was due to the abundant carboxylic groups in the PSG/SC system, which may consume a large amount of hydrogen ions for protonation [34]. Meanwhile, the weak repulsive forces between positively charged PSG/SC microcapsules may result in the formation of macromolecule aggregation [40]. Accordingly, the PSG/SC microcapsule solution appeared non-transparent. When the pH was above 5, the PSG/SC microcapsules were negatively charged. Increasing the pH value could enhance the absolute zeta potential. At pH 7, both PSG and SC carried sufficient negative charge, and the PSG/SC microcapsule solution became uniform and transparent. At this time, the strong electrostatic repulsive force between negatively charged macromolecules can prohibit coacervation, thus favoring the release of core material [41]. However, when pH was further increased to 8~9, the absolute zeta potential of PSG/SC microcapsules did not increase anymore. This is probably because the number of negatively charged functional groups has reached an apex, making it impossible to increase the absolute zeta potential.

#### 2.5.4. In Vitro Release

**Figure 8:**
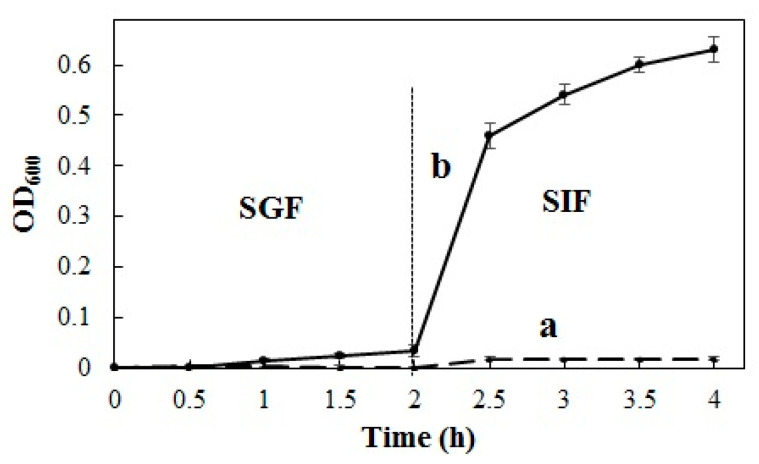
In vitro release profile of (a) empty PSG/SC microcapsules and (b) *L. plantarum*-loaded PSG/SC microcapsules in SGF and SIF (data shown were the mean ± SD, *n* = 3).

The in vitro release profile of PSG/SC microcapsules in SGF and SIF is shown in Figure 8. As can be seen, the release medium of empty PSG/SC microcapsules had a low absorbance over incubation time, with an OD_600_ of 0.004 at 2 h in SGF and 0.018 at 2 h in SIF, which could not affect the detection of *L. plantanum* release. The release amount of *L. plantarum* from microcapsules was minor during incubation in SGF, and OD_600_ was about 0.035 at 2 h. After the sample was transferred from SGF to SIF, the release of *L. plantarum* from microcapsules in SIF was much faster, and OD_600_ increased to 0.460 at 30 min in SIF. The initial release of SIF during the first 30 min was primarily due to the disintegration of the microcapsules. Then, the OD_600_ continuously increased and eventually reached 0.630, close to the original concentration of bacteria within 2 h, indicating the complete release of *L. plantarum*. The release behavior was consistent with the turbidity and zeta potential measurements. When PSG/SC microcapsules were transferred from SGF to SIF, -COOH groups were converted into -COO- groups, and both PSG and SC molecules were negatively charged. The electrostatic attraction between PSG and SC molecules almost disappeared; hence, the microcapsules began to disintegrate rapidly [42]. Furthermore, Jiang et al. indicated that the probiotic cultures were more sensitive to gastric conditions and tolerated intestinal conditions. Protein can form a more uniform and stable microstructure and is more resistant to gastric juice than alginate as a wall material. At the same time, alginate-based microcapsules were visually porous and much less uniform in structure [28]. Ma et al. also reported that microencapsulation in lactoprotein-protected *L. plantarum* LIP-1 was effectively compared with nonencapsulated cells in SGF and entirely released in SIF. This could be attributed to the density structure and the excellent protective buffering capacity of lactoprotein [30].

## 3. Materials and Methods

### 3.1. Materials

Pufferfish skin was provided by Fujian Puffer Aquaculture Co., Ltd. (Xiamen, China). A probiotic bacterium *L. plantarum* MCCC 1K05759 (DSM 20174) strain was provided by the Marine Culture Collection of China (Xiamen, China). Man Rogosa Sharpe (MRS) broth and agar were purchased from Solarbio Technology Co., Ltd. (Beijing, China). Soy protein isolate and whey protein of food grade were purchased by Linyi Shansong Biological Products Co., Ltd. (Linyi, China). Sodium caseinate of food grade was purchased from Henan Qianbo Chemical Products Co., Ltd. (Zhengzhou, China). Commercial bovine gelatin (BG) was purchased from Xilong Scientific Co., Ltd. (Shantou, China). Standard amino acids were purchased from Sigma company (St. Louis, MO, USA). Sterile simulated gastric fluid (SGF, pH 1.2) and intestinal fluid (SIF, pH 6.8) were obtained by Beijing Leagene Biotechnology Co., Ltd. (Beijing, China). All other chemicals were of analytical grade.

### 3.2. Preparation of Pufferfish Skin Gelatin

Pufferfish skin was first washed to remove impurities. A total of 1 Kg of clean pufferfish skin was stirred in 6 L of 0.2% NaOH solution for 2 h and subsequently in 6 L of 0.5% hydrochloric acid solution for 1 h. After alkaline and acid pretreatment, the swollen pufferfish skin was washed until neutral. The gelatin was extracted by adding 1 L of pure water and mechanical stirring at 60 °C for 4 h. Then, the gelatin solution was air-cooled and centrifuged at 12,000× *g* for 20 min using a CR22N high-speed refrigerated centrifuge (Hitachi, Japan) to remove the residue. The obtained gelatin extract (~30 g/L) was purified and concentrated by the 1812-type spiral-wound nanofiltration membrane with a molecular weight cut-off of 200 Da (Suntar Membrane Technology Co., Xiamen, China). The feed volume of nanofiltration was about 10 L. The whole nanofiltration process was conducted at room temperature with an operating pressure of 1.4 MPa. Finally, the purified gelatin was lyophilized by an FNLY-2 freeze drier (Fino Medical Equipment Co., Ltd., Shanghai, China) for 48 h. The moisture content of lyophilized gelatin was about 5.2%.

### 3.3. Bacterial Cultivation

*L. plantarum* was incubated in MRS broth at 37 °C for 14 h with agitation (120 r/min). *L. plantarum* was sub-cultured (37 °C, 14 h) twice in MRS broth for activation and adaptation. About 1 g (wet weight) of cells were obtained by centrifugation at 6760× *g* for 10 min from 100 mL of bacterial culture with a high-speed refrigerated centrifuge (CR22N, Hitachi, Japan). Fresh cells were subsequently subjected to microencapsulation. All the media and glassware used in this procedure were sterilized at 121 °C for 20 min.

### 3.4. Microencapsulation of L. plantarum 

Natural pH-dependent proteins like sodium caseinate (SC), soy protein isolate (SPI), and whey protein (WPI) were designed to compound with PSG as wall materials. A total of 10 g (wet weight) of fresh cells were mixed with 1 L of 20 g/L SC, SPI, or WPI solution. The cells were suspended using a vortex for about 10 min at room temperature. A total of 40 g/L PSG solution was then gently added, and the mixture was stirred for about 30 min. Finally, the mixture was spray dried by a laboratory scale spray dryer (SY-6000 spray dryer, Shiyuan, Shanghai, China), operating at a constant air inlet temperature of 120 °C and outlet temperature of 80 °C. In the spray-drying process, the mixture was continuously stirred at room temperature and pumped into the main chamber with a feed flow of 10 mL/min. After finishing the spray-drying, the microcapsules were obtained from the cyclone bottom and stored in sealed sterile vials at 4 °C.

### 3.5. Viable Count of L. plantarum Microcapsules

About 0.1 g of *L. plantarum* microcapsules were vortexed in 2 mL of filter-sterilized phosphate buffer (0.1 mol/L, pH 7.0) with 0.2 mol/L NaHCO_3_ until complete disintegration. Then, 50 μL of dissolved solution was added into 5 mL of sterile water for serial dilution, and 100 μL of appropriate diluted solution was uniformly mixed with MRS agar. Plate count in MRS agar numerated the viable cells after incubation at 37 °C for 48 h [43].

### 3.6. Survival Rate in Simulated Gastrointestinal Fluid

The sample (0.1 g) was mixed with 5 mL of pre-warmed SGF or SIF in a centrifuge tube and incubated for 120 min at 37 °C under 100 r/min [21]. Then, the sample was centrifuged at 4100× *g* for 5 min to separate cells from SGF or SIF and washed with sterile water twice. The survival rate was calculated based on the following equation:

survival rate (%) = N_1_/N × 100

where N is the viable count of *L. plantarum* in microcapsules untreated with SGF or SIF, and N_1_ is the viable count of *L. plantarum* in microcapsules treated with SGF or SIF.

### 3.7. Storage Experiment

To determine the storage stability of the *L. plantarum* microcapsules, 2 g of spray-dried sample was stored in sealed sterile penicillin vials at 4 °C with a relative humidity of 60 ± 10% for 6 months [44]. About 0.1 g of each sample was taken every month for viable count.

### 3.8. Turbidity Measurement

Turbidity measurements were performed by dispersing empty microcapsules in a buffer solution at pH 1~9 with a total concentration of 5 mg/mL. Turbidity assessments were performed using a UV–vis spectrophotometer (F-2700, Hitachi, Tokyo, Japan) at 600 nm with a glass cuvette (1 cm path length) at room temperature [45]. Deionized water was used as a blank for all solutions tested. The turbidity was calculated based on the following equation:

Turbidity (%) = (1 − T) × 100

where T is the transmittance of microcapsule solution at 600 nm.

### 3.9. Signal-Factor Experiment

The preparation conditions of *L. plantarum*-loaded PSG/SC microcapsules were optimized by signal-factor experiment. During the experiment, only one factor was changed, whereas the other two factors were fixed. The effects of PSG to SC mass ratio, SC concentration, and *L. plantarum* concentration on the encapsulation efficiency and survival rate of *L. plantarum* microcapsules in SGF were investigated [29]. The factors and levels of the single-factor experimental design are shown in Table 3. The remaining microencapsulation process was the same as described in Section 3.4.

### 3.10. Determination of Encapsulation Efficiency

A total of 0.1 g of *L. plantarum* microcapsules were vortexed in 4 mL of filter-sterilized phosphate buffer (0.1 mol/L, pH 7.0) with 0.2 mol/L NaHCO_3_ until complete disintegration to determine the total amount of *L. plantarum* in microcapsules. An equal-quality microcapsule sample was suspended in 4 mL of filter sterilized buffer solution (pH 5.0) at 37 °C for 45 min under 100 r/min, and the supernatant was collected to determine the viable count of non-microencapsulated *L. plantarum*. The encapsulation efficiency was calculated based on the following equation [46]:

encapsulation efficiency (%) = (N − N_0_)/N × 100

where N is the total viable count of both microencapsulated and non-microencapsulated *L. plantarum*, and N_0_ is the viable count of non-microencapsulated *L. plantarum*.

### 3.11. Preparation of L. plantarum-Loaded BG/SC Microcapsules

Under the conditions of a BG/SC mass ratio of 2:1, SC concentration of 20 g/L, and *L. plantarum* concentration of 10 g/L, *L. plantarum*-loaded BG/SC microcapsules were obtained by spray-drying. The preparation process was the same as described in Section 3.4.

### 3.12. Amino Acid Composition

A total of 0.1 g of gelatin was weighed and hydrolyzed by 10 mL of 6 mol/L hydrochloric acid at 110 °C for 24 h. Then, the sample solution was diluted 200 times and filtered with a 0.44 μm filter membrane. The amino acid composition of the sample was analyzed by Diane ICS 3000 ion chromatography (Thermo Fisher Scientific Inc, Waltham, MA, USA) with an amino acid analysis column AminoPacPA10 (2 mm × 250 mm) and a protection column AminoPacPA10 (2 mm × 50 mm). A total of 1 mol/L sodium acetate and 250 mmol/L NaOH solution were used as the mobile phase, and 10 μL of sample solution was loaded for analysis.

### 3.13. Zeta Potential Determination

Zeta potential was determined using a zeta potential analyzer (Nano-zs&MPT-2, Malvern Instruments, Worcestershire, UK). Empty microcapsules were dispersed in a Britton–Robinson buffer solution of different pH values at a 0.5 mg/mL concentration to measure zeta potential.

### 3.14. Morphology and Particle Size

The morphology was detected by a transmission electron microscope (TEM, JEM-2100, JEOL Ltd., Tokyo, Japan) and a scanning electron microscope (SEM, Quanta450, FEI Ltd., Portland, OR, USA). The sample was dispersed in ethanol at 2 mg/mL. Then, 1 μL of sample solution was pipetted to a copper grid and air-dried at room temperature for TEM characterization. For SEM characterization, the sample was placed on a piece of conductive adhesive and coated with gold by vacuum sputtering (PP3000T Cryo SEM System, Quorum Technologies Ltd., East Sussex, UK). Particle size distribution was measured by a particle size analyzer (LS-POP(9), Zhuhai Omec Instrument Co. Ltd., Zhuhai, China). About 60 mL of 1~2 mg/mL microcapsule solution was added into the sample cell, and the shading ratio was set at 10~15% for the test. 

### 3.15. Infrared

The Fourier transform infrared (FTIR) spectrum of the sample was performed using a Fourier transform infrared spectrometer (VER TEX70, Bruker Corporation, Karlsruhe, Germany). The samples were prepared by the KBr-disk method.

### 3.16. In Vitro Release Studies

A total of 0.1 g of *L. plantarum*-loaded PSG/SC microcapsules were added to 20 mL of SGF, incubated at 37 °C for 2 h under orbital shaking at 100 r/min, and subsequently transferred into SIF for another 2 h. Empty PSG/SC microcapsules were used as a control. About 0.2 mL of the upper release medium was taken for detection at specific time intervals (30 min) and replaced by fresh medium at the same time [47]. OD_600_ of each sample for *L. plantarum* was assayed in triplicate using a UV–vis spectrophotometer (F-2700, Hitachi, Tokyo, Japan) to assess release [48].

### 3.17. Statistical Analysis

All the experiments were carried out in triplicate. The original data was averaged and processed using Excel 2013 or SPSS 16.0. An analysis of variance (ANOVA) was used to evaluate the significant difference between the mean values. The significance level of *p* < 0.05 was used throughout the study.

## 4. Conclusions

PSG-based wall materials were used to encapsulate *L. plantarum* by spray-drying. When SC was combined with PSG, the microcapsules provided better protection for *L. plantarum* than SPI and WPI in spray-drying, simulated digestion, and storage. The turbidity and zeta potential characteristics of PSG/SC at a mass ratio of 2:1 at different pH levels suggested that it was suitable as a gastrointestinal delivery carrier for probiotics. Gelatin species also had a significant effect on microcapsule performance. Compared with PSG/SC microcapsules, BG/SC microcapsules had a lower encapsulation efficiency and survival rate in simulated digestion. This may be due to the much higher imine and aspartic acid content of PSG, as well as the unique surface charge characteristic of PSG/SC. Both TEM and FTIR analyses indicated that *L. plantarum* was well embedded in PSG/SC microcapsules. PSG/SC microcapsules did not disintegrate in SGF (2 h) but could be completely released in SIF (2 h), allowing probiotics to resist the stress of digestive fluid. Therefore, PSG/SC was a prospective carrier for the encapsulation of bacteria and could be potentially employed in the food and pharmaceutical fields.

## Figures and Tables

**Table 1:** Effect of wall materials on the viability of *L. plantarum* during spray-drying.

Wall Material	Viability of *L. plantarum* (log CFU/g)	
	Before Spray-Drying	After Spray-Drying
None	9.0 ± 0.1	2.1 ± 0.2
PSG/SC	9.0 ± 0.2	7.7 ± 0.1
PSG/SPI	8.9 ± 0.3	5.2 ± 0.2
PSG/WPI	9.0 ± 0.1	6.9 ± 0.1

Values are presented as the mean value ± standard deviation (SD) from triplicate experiments.

**Table 2:** Amino acid composition of pufferfish skin gelatin and commercial bovine gelatin.

Amino Acids	Values (g/100 g Protein)	
	Pufferfish Skin	Commercial Bovine
Aspartic acid (Asp)	26.16	6.67
Threonine (Thr)	1.25	2.05
Serine (Ser)	0.97	3.75
Glutamic acid (Glu)	9.27	9.89
Glycine (Gly)	13.67	19.65
Alanine (Ala)	7.17	7.58
Cystine (Cys)	0.21	1.15
Valine (Val)	0.90	2.81
Methionine (Met)	0.50	0.93
Isoleucine (Ile)	0.63	1.45
Leucine (Leu)	0.69	3.08
Tyrosine (Tyr)	0.17	0.72
Phenylalanine (Phe)	0.96	2.39
Histidine (His)	1.39	0.97
Lysine (Lys)	1.11	4.93
Arginine (Arg)	6.97	8.51
Proline (Pro)	3.89	13.36
Hydroxyproline (Hyp)	24.09	10.11
Total	100.00	100.00

**Table 3:** Factors and levels of single-factor experiment.

Assay	PSG/SC Mass Ratio	SC Concentration (g/L)	*L. plantarum* Concentration (g/L)
Effect of PSG/SC mass ratio	1:2	10	10
	1:1		
	2:1		
	3:1		
	4:1		
Effect of SC concentration	2:1	5	10
		10	
		20	
		30	
		40	
Effect of *L. plantarum* concentration	2:1	20	5
			10
			20
			30
			40

## Data Availability

The original data presented in the study are included in the article; further inquiries can be directed to the corresponding author.

## References

[1] Morelli L., Pellegrino P. (2021). A critical evaluation of the factors affecting the survival and persistence of beneficial bacteria in healthy adults. Benef. Microbes.

[2] Parsana Y., Yadav M., Kumar S. (2023). Microencapsulation in the chitosan-coated alginate-inulin matrix of *Limosilactobacillus reuteri* SW23 and *Lactobacillus salivarius* RBL50 and their characterization. Carbohydr. Polym. Technol. Appl..

[3] Fraj J., Petrović L., Đekić L., Budinčić J.M., Bučko S., Katona J. (2021). Encapsulation and release of vitamin C in double W/O/W emulsions followed by complex coacervation in gelatin-sodium caseinate system. J. Food Eng..

[4] Luca L., Oroian M. (2021). Influence of different prebiotics on viability of *Lactobacillus casei*, *Lactobacillus plantarum* and *Lactobacillus rhamnosus* encapsulated in alginate microcapsules. Foods.

[5] Sun W., Nguyen Q.D., Sipiczki G., Ziane S.R., Hristovski K., Friedrich L., Visy A., Hitka G., Gere A., Bujna E. (2023). Microencapsulation of *Lactobacillus plantarum* 299v strain with whey proteins by lyophilization and its application in production of probiotic apple juices. Appl. Sci..

[6] Xu Y., Dong M., Xiao H., Quek S.Y., Ogawa Y., Ma G., Zhang C. (2023). Advances in spray-dried probiotic microcapsules for targeted delivery: A review. Crit. Rev. Food Sci. Nutr..

[7] Avila-Reyes S.V., Garcia-Suarez F.J., Jiménez M.T., Martín-Gonzalez M.F.S., Bello-Perez L.A. (2014). Protection of *L. rhamnosus* by spray-drying using two prebioticscolloids to enhance the viability. Carbohydr. Polym..

[8] Du T., Liu Z., Guan Q., Xiong T., Peng F. (2023). Application of soy protein isolate–xylose conjugates for improving the viability and stability of probiotics microencapsulated by spray drying. J. Sci. Food Agric..

[9] Ozdemir N., Bayrak A., Tat T., Altay F., Kiralan M., Kurt A. (2021). Microencapsulation of basil essential oil: Utilization of gum arabic/whey protein isolate/maltodextrin combinations for encapsulation efficiency and in vitro release. J. Food Meas. Charact..

[10] Peñalva R., Martínez-López A.L., Gamazo C., Gonzalez-Navarro C., González-Ferrero C., Virto-Resano R., Brotons-Canto A., Vitas A., Collantes M., Peñuelas I. (2023). Encapsulation of *Lactobacillus plantarum* in casein-chitosan microparticles facilitates the arrival to the colon and develops an immunomodulatory effect. Food Hydrocoll..

[11] Vaziri A.S., Alemzadeh I., Vossoughi M., Khorasani A.C. (2018). Co-microencapsulation of *Lactobacillus plantarum* and DHA fatty acid in alginate-pectin-gelatin biocomposites. Carbohydr. Polym..

[12] Bastos B.M., Farias B.S., Casati M.O., Engelmann J.I., Moura J.M., Pinto L.A.A. (2021). Gelatin films from carp skin crosslinked by gallic acid and incorporated with chitosan/tuna lipid fractions. J. Polym. Environ..

[13] Pan J.F., Lian H.L., Shang M., Jin W., Hao R., Ning Y., Zhang X., Tang Y. (2020). Physicochemical properties of Chinese giant salamander (*Andrias davidianus*) skin gelatin as affected by extraction temperature and in comparison with fish and bovine gelatin. J. Food Meas. Charact..

[14] Derkach S.R., Voron’ko N.G., Kuchina Y.A., Kolotova D.S. (2020). Modified fish gelatin as an alternative to mammalian gelatin in modern food technologies. Polymers.

[15] Pan J., Li Q., Jia H., Xia L., Jin W., Shang M., Xu C., Dong X. (2018). Physiochemical and functional properties of tiger puffer (*Takifugurubripes*) skin gelatin as affected by extraction conditions. Int. J. Biol. Macromol..

[16] Wang S., Zhou D., Liu N., Sun Y., Sun G. (2023). Physicochemical and fibril formation properties of pufferfish (*Takifugu obscurus*) skin collagen from solvent extraction in different conditions. Gels.

[17] Wang Z., Wu J., Tian Z., Si Y., Chen H., Gan J. (2022). The Mechanisms of the potential probiotic *Lactiplantibacillus plantarum* against cardiovascular disease and the recent developments in its fermented foods. Foods.

[18] Hao R., Chen Z., Wu Y., Li D., Qi B., Lin C., Zhao L., Xiao T., Zhang K., Wu J. (2024). Improving the survival of *Lactobacillus plantarum* FZU3013 by phase separated caseinate/alginate gel beads. Int. J. Biol. Macromol..

[19] Guo Q., Li S., Tang J., Chang S., Qiang L., Du G., Yue T., Yuan Y. (2022). Microencapsulation of *Lactobacillus plantarum* by spray drying: Protective effects during simulated food processing, gastrointestinal conditions, and in kefir. Int. J. Biol. Macromol..

[20] Moumita S., Das B., Hasan U., Jayabalan R. (2018). Effect of long-term storage on viability and acceptability of lyophilized and spray-dried synbiotic microcapsules in dry functional food formulations. LWT-Food Sci. Technol..

[21] Arslan S., Erbas M., Tontul I., Topuz A. (2015). Microencapsulation of probiotic *Saccharomyces cerevisiae* var. boulardii with different wall materials by spray drying. LWT-Food Sci. Technol..

[22] Guo H.H., Chen H., Yun Y., Fang H., Chen S., Hong Z. (2020). Study on the composition and acute toxicity of collagen peptide chelated zinc from puffer skin. Food Ferment. Ind..

[23] Zhao M., Huang X., Zhang H., Zhang Y., Gänzle M., Yang N., Nishinari K., Fang Y. (2020). Probiotic encapsulation in water-in-water emulsion via heteroprotein complex coacervation of type-A gelatin/sodium caseinate. Food Hydrocoll..

[24] Duhoranimana E., Karangwa E., Lai L., Xu X., Yu J., Xia S., Zhang X., Muhoza B., Habinshuti I. (2017). Effect of sodium carboxymethyl cellulose on complex coacervates formation with gelatin: Coacervates characterization, stabilization and formation mechanism. Food Hydrocoll..

[25] Kavousi H.R., Fathi M., Goli S. (2018). Novel cress seed mucilage and sodium caseinatemicroparticles for encapsulation of curcumin: An approach for controlled release. Food Bioprod. Process..

[26] Ramos P.E., Cerqueira M.A., Cook M.T., Bourbon A.I., Khutoryanskiy V.V., Charalampoulos D., Teixeira J.A., Vicente A.A. (2016). Development of an immobilization system for in situ micronutrients release. Food Res. Int..

[27] Dehkordi S.S., Alemzadeh I., Vaziri A.S., Vossoughi A. (2020). Optimization of alginate-whey protein isolate microcapsules for survivability and release behavior of probiotic bacteria. Appl. Biochem. Biotechnol..

[28] Jiang Y., Zheng Z., Zhang T., Hendricks G., Guo M. (2016). Microencapsulation of Lactobacillus acidophilus NCFM using polymerized whey proteins as wall material. Int. J. Food Sci. Nutr..

[29] Sun Q., Wang F., Han D., Zhao Y., Liu Z., Lei H., Yang Z. (2014). Preparation and optimization of soy protein isolate–high methoxy pectin microcapsules loaded with Lactobacillus delbrueckii. Int. J. Food Sci. Technol..

[30] Ma L., Shang Y., Zhu Y., Zhang X., E J., Zhao L., Wang J. (2020). Study on microencapsulation of Lactobacillus plantarum LIP-1 by emulsification method. J. Food Process Eng..

[31] Gómez-Guillén M.C., Giménez B., López-Caballero M.E., Montero M.P. (2011). Functional and bioactive properties of collagen and gelatin from alternative sources: A review. Food Hydrocoll..

[32] Pavan Kumar D., Chandra M.V., Elavarasan K., Shamasundar B.A. (2017). Structural properties of gelatin extracted from croaker fish (Johnius sp) skin waste. Int. J. Food Prop..

[33] Kusumaningrum I., Pranoto Y., Hadiwiyoto S. (2018). Extraction optimization and characterization of gelatine from fish dry skin of Spanish mackerel (*Scomberromorus commersoni*). IOP Conf. Ser. Earth Environ. Sci..

[34] Roy J.C., Giraud S., Ferri A., Mossotti R., Guan J., Salaün F. (2018). Influence of process parameters on microcapsule formation from chitosan—Type B gelatin complex coacervates. Carbohydr. Polym..

[35] Liu H., Gong J., Chabot D., Miller S.S., Cui S.W., Ma J., Zhong F., Wang Q. (2015). Protection of heat-sensitive probiotic bacteria during spray-drying by sodium caseinate stabilized fat particles. Food Hydrocoll..

[36] Bhagwat A., Bhushette P., Annapure U.S. (2020). Spray drying studies of probiotic Enterococcus strains encapsulated with whey protein and maltodextrin. Beni-Suef Univ. J. Basic Appl. Sci..

[37] De Castro-Cislaghi F.P., E Silva C.D.R., Fritzen-Freire C.B., Lorenz J.G., Sant’Anna E.S. (2012). Bifidobacterium Bb-12 microencapsulated by spray drying with whey: Survival under simulated gastrointestinal conditions, tolerance to NaCl, and viability during storage. J. Food Eng..

[38] Huang X., Gänzle M., Zhang H., Zhao M., Fang Y., Nishinari K. (2021). Microencapsulation of probiotic lactobacilli with shellac as moisture barrier and to allow controlled release. J. Sci. Food Agric..

[39] Shaharuddin S., Muhamad I.I. (2015). Microencapsulation of alginate-immobilized bagasse with Lactobacillus rhamnosus NRRL 442: Enhancement of survivability and thermotolerance. Carbohydr. Polym..

[40] Karagozlu M., Ocak B., Özdestan-Ocak Ö. (2021). Effect of Tannic Acid Concentration on the Physicochemical, Thermal, and Antioxidant Properties of Gelatin/Gum Arabic–Walled Microcapsules Containing *Origanum onites* L. Essential Oil. Food Bioprocess Technol..

[41] Shaddela R., Hesaria J., Azadmard-Damirchia S., Hamishehkar H., Fathi-Achachlouei B., Huang Q. (2018). Use of gelatin and gum Arabic for encapsulation of black raspberryanthocyanins by complex coacervation. Int. J. Biol. Macromol..

[42] Li X.Y., Chen X.G., Cha D.S., Park H.J., Liu C.S. (2009). Microencapsulation of a probiotic bacteria with alginate–gelatin and its properties. J. Microencapsul..

[43] Nag A., Han K., Singh H. (2011). Microencapsulation of probiotic bacteria using pH-induced gelation of sodium caseinate and gellan gum. Int. Dairy J..

[44] Liao L., Wei X., Gong X., Li J., Huang T., Xiong T. (2017). Microencapsulation of Lactobacillus casei LK-1 by spray drying related to its stability and in vitro digestion. LWT-Food Sci. Technol..

[45] Camargo T.R., Khelissa S., Chihib N.E., Dumas E., Wang J., Valenti W.C., Gharsallaoui A. (2021). Preparation and characterization of microcapsules containing antioxidant fish protein hydrolysates: A new use of bycatch in brazil. Mar. Biotechnol..

[46] Würth R., Wiesner S., Foerst P., Kulozik U. (2017). Impact of the CaCl_2_ content in the rehydration media on the microcapsule formation out of spray dried capsule precursors for the immobilization of probiotic bacteria. Int. Dairy J..

[47] Iqbal R., Zahoor T., Huma N., Jamil A., Ünlü G. (2019). In-vitro GIT Tolerance of microencapsulated bifidobacterium bifidum ATCC 35914 using polysaccharide-protein matrix. Probiotics Antimicrob. Proteins.

[48] Su Y., Zheng X., Zhao Q., Fu N., Xiong H., Wu W.D., Chen X.D. (2019). Spray drying of Lactobacillus rhamnosus GG with calcium-containing protectant for enhanced viability. Powder Technol..

